# Co-administered antibody improves penetration of antibody–dye conjugate into human cancers with implications for antibody–drug conjugates

**DOI:** 10.1038/s41467-020-19498-y

**Published:** 2020-11-09

**Authors:** Guolan Lu, Naoki Nishio, Nynke S. van den Berg, Brock A. Martin, Shayan Fakurnejad, Stan van Keulen, Alexander D. Colevas, Greg M. Thurber, Eben L. Rosenthal

**Affiliations:** 1 Department of Otolaryngology – Head and Neck Surgery, Stanford University School of Medicine, 900 Blake Wilbur Drive, Stanford, CA 94305 USA; 2 Department of Otorhinolaryngology, Nagoya University Graduate School of Medicine, 65 Tsurumai-cho, Showa-ku, Nagoya, 466-8550 Japan; 3 Department of Pathology, Stanford University School of Medicine, 300 Pasteur Drive, Stanford, CA 94305 USA; 4 Department of Medicine, Division of Oncology, Stanford University School of Medicine, 875 Blake Wilbur Drive, Stanford, CA 94305 USA; 5 Department of Chemical Engineering, University of Michigan, Ann Arbor, MI USA; 6 Department of Biomedical Engineering, University of Michigan, Ann Arbor, MI USA

**Keywords:** Cancer imaging, Head and neck cancer, Drug delivery, Phase I trials

## Abstract

Poor tissue penetration remains a major challenge for antibody-based therapeutics of solid tumors, but proper dosing can improve the tissue penetration and thus therapeutic efficacy of these biologics. Due to dose-limiting toxicity of the small molecule payload, antibody-drug conjugates (ADCs) are administered at a much lower dose than their parent antibodies, which further reduces tissue penetration. We conducted an early-phase clinical trial (NCT02415881) and previously reported the safety of an antibody-dye conjugate (panitumumab-IRDye800CW) as primary outcome. Here, we report a retrospective exploratory analysis of the trial to evaluate whether co-administration of an unconjugated antibody could improve the intratumoral distribution of the antibody-dye conjugate in patients. By measuring the multiscale distribution of the antibody-dye conjugate, this study demonstrates improved microscopic antibody distribution without increasing uptake (toxicity) in healthy tissue when co-administered with the parent antibody, supporting further clinical investigation of the co-administration dosing strategy to improve the tumor penetration of ADCs.

## Abstract (web summary)

Antibody-drug conjugates targeting high expression receptors can suffer from poor tumour penetration. Here, the authors use unconjugated antibody to improve the penetration of an antibody-dye conjugate in a clinical study, supporting further clinical investigation of the co-administration strategy.

## Introduction

Antibody-based therapeutics comprise the largest portion of the growing number of anticancer drugs. However, poor tissue penetration remains a major hurdle limiting the efficacy of these biologics^1–4^. Proper dosing of antibody-based biologics is an important factor in improving tissue penetration and thus therapeutic efficacy for treating solid tumors^5,6^. Antibody–drug conjugates (ADCs), which combine the antigen specificity of antibody and the potency of cytotoxic agents, are an emerging class of antibody bioconjugates that could benefit from improved tissue penetration^7,8^. Owing to the dose-limiting toxicities of the potent cytotoxic payload, ADCs are usually administered at a much lower dose with narrower therapeutic window compared to their parent antibodies, which may result in decreased penetration into solid tumors. Even with serial dosing, the continuous internalization of the ADC can prevent delivery to all cells^9^. To date, ~100 ADCs are being tested in over 400 clinical trials. Of the nine FDA approved ADCs, ado-trastuzumab emtansine (T-DM1) was the only approved agent for solid tumors until recently (with enfortumab vedotin-ejfv, fam-trastuzumab-deruxtecan-nxki, and sacituzumab govitecan-hziy gaining approval between December 2019 and April 2020), whereas the remaining agents treat hematological malignancies. Dosing strategies that maximize tumor penetration and minimize delivery to off-target tissue (toxicity) could expand the therapeutic window of ADCs and improve their therapeutic benefit.

There is clinical precedence that manipulating dosing strategies can mitigate ADC toxicity. For example, administration of a lower dose of gemtuzumab ozogamicin at a higher frequency produces less toxicity and improves tolerability, such as lower thrombocytopenia and fewer abnormal liver function indicators^10^. In another clinical trial, reducing the weekly dosing of coltuximab ravtansine to once every 2 weeks lowered the toxicity to nervous system and ocular toxicities^11^. Although reducing the dose and increasing dosing frequency improves tolerability^12^, the lower plasma concentrations can result in lower tumor uptake, which affects therapeutic results^6^. Furthermore, intratumoral drug distribution remains unknown since changes to dosing strategies are typically only measured from plasma that does not necessarily reflect intratumoral drug levels^5^.

An alternative ADC-dosing strategy proposes that co-administration of the parent or unlabeled antibody (loading dose or LD) with the ADC compound could overcome the binding site barrier of antibodies and thus improve the distribution of ADCs within the tumor^7,13^. In preclinical studies, trastuzumab has been administered with a therapeutic dose of T-DM1 at a ratio of 0:1, 1:1, 3:1, and 8:1 into tumor-bearing mice. Using the co-administration strategies, T-DM1 penetrated beyond the perivascular region to tumor areas further away from the vasculature, and the improved penetration also resulted in better therapeutic efficacies. Moreover, the benefit of this dosing strategy in high-antigen expression tumors was confirmed in a more recent preclinical study^14^. However, as a mouse model cannot recapitulate the complexity of human tumors, the clinical utility of this dosing strategy remains unknown. Clinical validation is critical to elucidate the potential impact of a loading dose and to quantify, to what extent, if any, it improves the tissue penetration. If this dosing strategy works in human patients, this could have a major impact on the ADC development given the dose-limiting toxicities of these agents and the number of clinical trials on ADCs.

Although poor tissue penetration has been recognized as a bottleneck for antibody-based therapeutics for decades, it has only recently been characterized with cellular resolution in human tumors by our group, using a near-infrared (NIR) fluorescence-labeled antibody (panitumumab-IRDye800CW) as an imaging tool^2^. This early-phase clinical trial (NCT02415881) provided a suitable dataset to test the preclinical hypothesis that a loading dose of antibody could improve the microscopic distribution of the antibody–dye conjugate in a clinical setting. The primary outcome of this trial was pre-defined as the safety profile of panitumumab-IRDye800CW and was previously reported^15^. Here, we report a retrospective ad hoc analysis of the clinical trial to evaluate the co-administration dosing strategy in the clinic. We hypothesize that the fluorescently labeled antibody might be used as a surrogate to measure the intratumoral distribution of ADCs. The rationale is that as long as the fluorophore or the cytotoxic drug is conjugated appropriately (not dramatically change the binding site or physicochemical properties), it has minimal influence on the pharmacokinetics and tissue penetration of the antibody. Our previous studies have demonstrated that the pharmacokinetics and toxicity of antibody–dye conjugates (i.e., panitumumab-IRDye800CW and cetuximab-IRDye800CW) mirror the parent antibody in macaques and human patients^15,16^. It has also been reported that proper conjugation of a parent antibody without too many drug payloads resulted in a similar pharmacokinetics to the parent antibody^17^. In the current study, we comprehensively evaluate the impact of co-administering the unconjugated antibody on the uptake and distribution of the antibody–dye conjugate at both the macroscopic and microscopic levels in clinical tumors. This study demonstrates improved intratumoral distribution of the antibody–dye conjugate with lower uptake in healthy tissue when co-administered with a loading dose of the parent antibody, which support the possibility of applying the co-administration dosing strategy to ADCs to expand their therapeutic window in solid tumors in the clinic.

## Results

### Summary of the study design

A single-center, non-randomized, phase I study was performed in 24 patients with head and neck squamous cell carcinomas (HNSCC) that received a total antibody dose of 0.3 mg kg^−1^ to 2.6 mg kg^−1^. Half of these patients received a loading dose of 100 mg unlabeled antibody (panitumumab). Patient characteristics are summarized in Table 1. Patient age ranged from 32 to 85 years old at diagnosis (mean: 62 years old), and the majority presented with oral squamous cell carcinoma (88%). The average time of infusion to the start of surgery was 2.5 days (range 1–5). No significant differences were found when comparing age, primary tumor site, tumor size and time of infusion-surgery between patients who received a loading dose (LD group) and those who did not (non-LD group).

**Table 1:** Patient characteristics.

Variable		LD group	Non-LD group	*p*
		*n* = 12	*n* = 12	
Age (years)	≤60	6	7	ns
	>60	6	5	
Gender	Male	11	6	*
	Female	1	6	
Primary site	Tongue	3	5	ns
	Other	9	7	
Tumor size (mm)	≤40	7	8	ns
	>40	5	4	
Time of infusion-surgery (days)	≤2	8	10	ns
	>3	4	2	

**Fig. 1:**
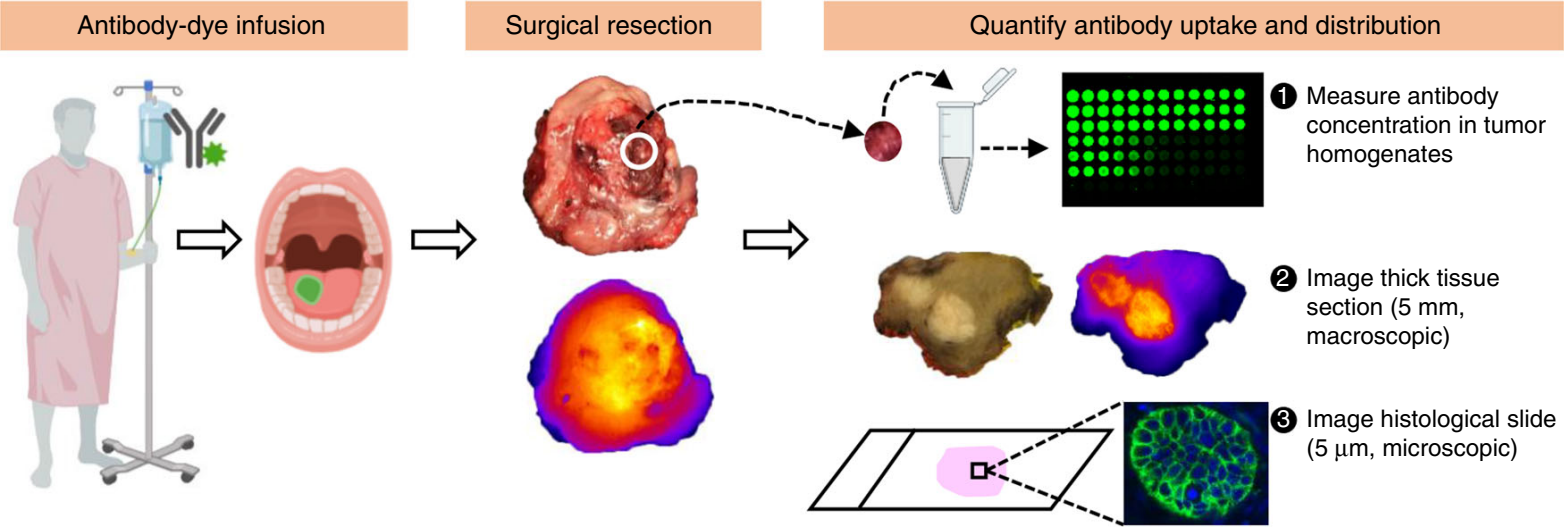
Overview of the study design. Study patients received infusion of antibody–dye conjugate with or without a loading dose of unlabeled antibody. One to five days post infusion, patients underwent surgical tumor resection. Fresh tissue samples obtained from the primary tumor were homogenized to quantify antibody–dye concentration in the tissue. Whole-tumor specimen were then formalin-fixed and cut into 5 mm thick tissue sections for macroscopic imaging. Subsequently, 5 µm histological slides were prepared from each tissue paraffin block made from the 5 mm tissue sections for microscopic imaging. Antibody uptake and distribution were measured from tissue homogenates, macroscopic, and microscopic imaging. Some elements of this figure were created with BioRender.com.

Panitumumab-IRDye800CW in primary tumors was measured using fresh tumor samples, thick tissue sections (5 mm), and histological slides (5 µm) (Fig. 1). Immediately after surgical resection, fresh tissue samples were obtained from each patient when available and antibody concentration was quantified using these tissue samples following a previously developed protocol^2^. After formalin-fixation, the primary tumor specimen was breadloafed into 5 mm thick tissue sections and imaged with a closed-field fluorescence imaging system. Subsequently, the thick tissue sections were paraffin embedded into tissue blocks and then serially sectioned at 5 µm thickness (histology sections) for NIR microscopic imaging and histopathology assessment (hematoxylin and eosin (H&E) slides). Antibody–dye uptake and distribution, as measured by fluorescence intensity and distribution, were quantified from both the thick tissue sections (macroscopic imaging) and the thin histological slides (microscopic imaging).

### Lower healthy tissue uptake (toxicity) with loading dose

**Fig. 2:**
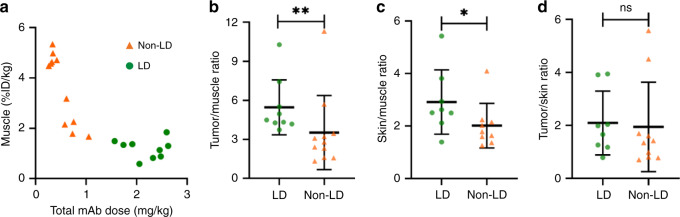
Co-administration of unlabeled antibody reduced muscle uptake while maintained tumor uptake. **a** Muscle antibody uptake (%ID kg^−^^1^: percent injected dose per kilogram) decreased with increasing total monoclonal antibody (mAb) dose (owing to loading dose (LD)). **b**, **c**. Tumor and skin uptake (%ID kg^−^^1^) as normalized by muscle uptake (%ID kg^−^^1^) were significantly higher in the loading dose group than that in the non-loading dose group, suggesting that tumor and skin were not fully saturated within the total antibody dose range of 0.3 mg kg^−1 ^to 2.6 mg kg^−1^. **d** When tumor uptake was normalized by skin uptake, there was no significant differences between groups. The number of independent patient samples available in **b**: *n* = 9 (LD), *n* = 11 (non-LD); **c** *n* = 8 (LD), *n* = 9 (non-LD); **d** *n* = 8 (LD), *n* = 10 (non-LD). *p* value = 0.010 **b**, 0.046 **c**. Mann–Whitney *U* test (two-tailed) were used for comparison in **b**–**d**. Graphs plotted mean with standard deviation. (*p < 0.05. **p < 0.01, ns: *p* > 0.05).

When the total antibody dose increased from 0.3 mg kg^−1^ to 2.6 mg kg^−1^, muscle uptake of the antibody–dye conjugate (%ID kg^−1^) showed a decreasing trend (Fig. 2a), suggesting that co-administration of unlabeled antibody saturates the estimated glomerular filtration rate (EGFR)-binding sites in the muscle and thus reduces the target-mediated uptake (relevant for ADC toxicity) in healthy tissue. To avoid the confounding effect of patient variability, we normalized the tumor and skin uptake by the muscle uptake, and found significantly higher uptake of antibody–dye in both tumor and skin tumors from patients receiving the loading dose (Fig. 2b, c). When tumor uptake was normalized by skin uptake, there was no significant differences between groups (Fig. 2d). Taken together, this data suggests that EGFR receptors were not saturated in either the skin or tumor.

### Heterogeneity of intratumoral antibody–dye distribution

**Fig. 3:**
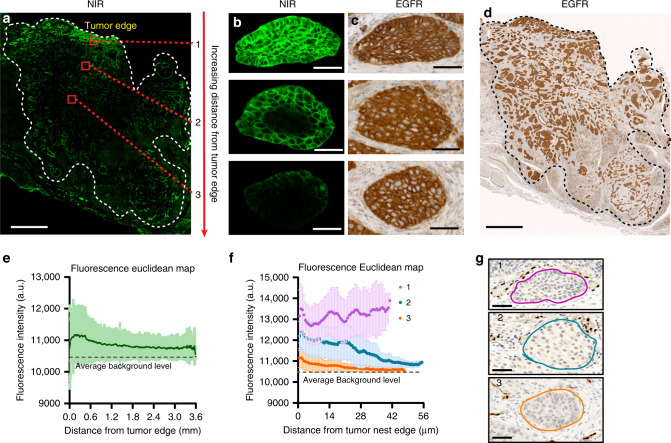
Antibody–dye distribution demonstrated both macroscopic and microscopic heterogeneity within the solid tumor. **a** Antibody–dye uptake (NIR: near-infrared) was higher in the tumor periphery or edge (especially the epithelium) than that in the tumor interior (Scale bar: 2 mm). **b**, **c** Antibody penetrated deeper into tumor nests located close to the edge of the bulk tumor than tumor nests located further away from the tumor edge, which was not attributable to EGFR (epidermal growth factor receptor) expression (Scale bar: 50 μm). **d** EGFR expression level is similar across the entire tumor (Scale bar: 2 mm). **e** The fluorescence intensity negatively correlated with increasing distance from the edge of the bulk tumor. **f** The fluorescence intensity plot depicted varying degree of antibody penetration into tumor nests: 1. fully saturated tumor nests at the periphery; 2. partially saturated tumor nests; 3. poorly penetrated tumor nests at the center of the tumor. (The shaded area in **e** and **f** means standard deviation). *n* = 3 tumor nests from the same patient. **g** Vessel staining (ERG) corresponding to region 1, 2, 3 in **f** (Scale bar: 50 μm).

Sub-saturating doses of systemic antibody resulted in heterogeneous distribution of the antibody–dye conjugate within the entire tumor mass and microscopically within tumor nests (Fig. 3). In large tumors, antibody delivery to the tumor interior was substantially lower than the periphery (Fig. 3a), despite the homogenous EGFR expression across the tumor (Fig. 3d). The fluorescence intensity plot (Fig. 3e) showed a slowly decreasing trend as the tumor cells were further away from the edge of the tumor mass, which was previously shown to be correlated with the higher vessel density and lower stromal density at the perphery^2^, and elevated interstitial pressure in the interior^18^. At the microscopic level, we noted varying degrees of antibody diffusion into the tumor nests despite consistent EGFR expression (Fig. 3b, c). The saturation of the tumor nests depended on their location relative to the edge of the tumor mass. At sub-saturating doses (consistent with ADC dosing), the peripheral tumor nests showed constant fluorescence intensity across the tumor nest, whereas more centrally located tumor nests had gradually decreasing fluorescence as distance from the edge of the tumor nests increased (Fig. 3f). The negative correlation between drug delivery and distance from the tumor edge at the microscopic and macroscopic level suggests that antibody first saturates peripherally located receptors. Although distribution is clearly heterogeneous, the distinct pattern of antibody penetration into tumors at the microscopic scale supports the development of a stalled saturation front in tumors due to plasma clearance and/or local tumor cell consumption^9^, often referred to as the “binding site barrier”^19^.

### Differential distribution in micro not macroscopic imaging

**Fig. 4:**
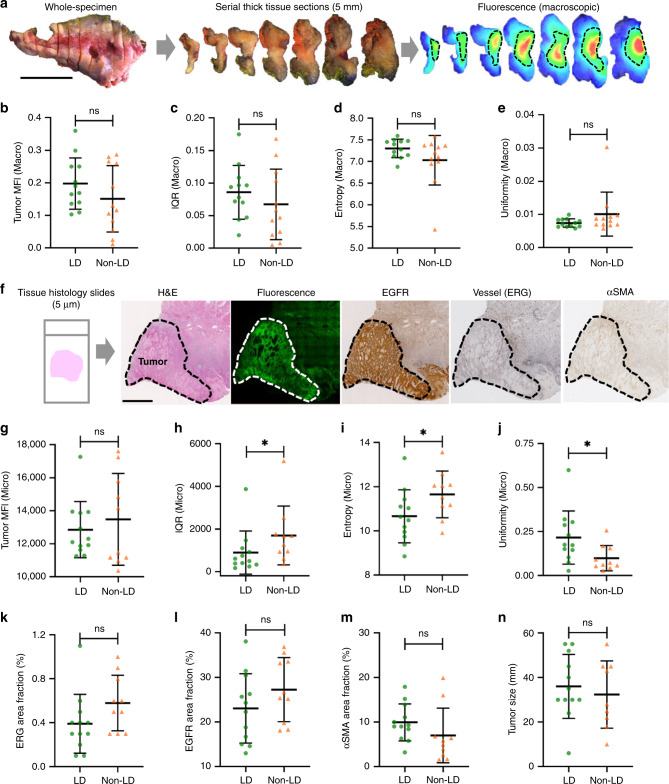
A loading dose of unlabeled antibody significantly reduced heterogeneity of antibody distribution in tumors, which can be captured by microscopic imaging but not macroscopic imaging. **a** Schematic workflow of reconstructing the whole-tumor fluorescence distribution and measuring antibody delivery by macroscopic imaging. **b**–**e** The tumor uptake as measured by the MFI, and the tumor antibody distribution quantified by IQR,entropy, and uniformity from macroscopic imaging showed no significant difference between the two dosing groups. **b**–**e** Tissue specimens were available in *n* = 12 patients in the LD group and *n* = 12 in the non-LD group. **f** Schematic workflow of measuring antibody delivery in tissue histological sections and tumor microenvironmental factors using microscopic imaging. **g**–**j** The tumor uptake measured by the MFI from microscopic imaging also showed no difference between the two groups, but the antibody distribution measured by IQR, entropy, and uniformity from microscopic imaging showed significantly higher heterogeneity in the non-LD group (*p < 0.05, ns: *p* > 0.05). *p* value = 0.030 **h**, 0.036 **i**, 0.025 **j**. **k**–**n** Vessel area fraction, EGFR area fraction, αSMA area fraction, and tumor size were not statistically different between the LD and the non-LD group. (Scale bar in **a** and **f**: 2 cm; graphs plotted mean with standard deviation). **g**–**n** Tissue specimens were available in *n* = 12 patients in LD group and *n* = 10 in the non-LD group. Mann–Whitney *U* test (two-tailed) was used in **b**–**e**, **g**–**n**.

After whole-tumor reconstruction using individual 5 mm thick tissue sections (Fig. 4a), we were able to quantify the fluorescence distribution over the entire tumor using macroscopic imaging. We found that the tumor mean fluorescence intensity (MFI), interquartile range (IQR), entropy, and uniformity in the LD group were similar to those in the non-LD group (Fig. 4b–e). Next, we analyzed fluorescence distribution from the microscopic imaging of 5 µm histological slides (Fig. 4f). Although tumor MFI in the LD were similar to that in the non-LD group, the IQR and entropy of fluorescence signal over the tumor area of histology slides was significantly lower in the LD group than the non-LD group, whereas the uniformity of the LD group was significantly higher than that of the non-LD group (Fig. 4g–j). These data suggested that co-administration of unlabeled antibody did not decrease the uptake of antibody–dye conjugate, but rather improved the homogeneity of antibody distribution across the tumor, which can only be measured by microscopic imaging but not the macroscopic imaging. Furthermore, factors that we and others have correlated with antibody delivery, including the vessel area fraction, EGFR area fraction, α-smooth muscle actin (αSMA) area fraction (activated stromal density), and tumor size, were not significantly different between the two groups (Fig. 4k–n). Thus the differential distribution between the two dosing groups were likely not caused by differences in vessel, EGFR, αSMA, or tumor size.

### Loading dose improved the antibody penetration in the tumor

**Fig. 5:**
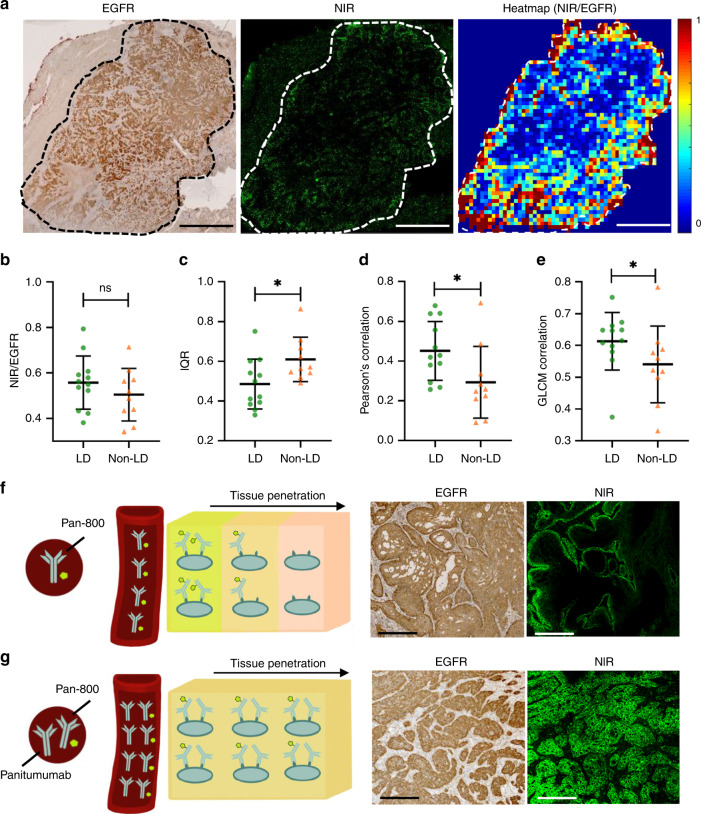
Antibody–dye has more homogenous spatial distribution across tumor when co-administered with the unlabeled antibody. **a** Each tumor was gridded into small square regions and antibody penetration within each square region was quantified as the ratio of fluorescent positive area to EGFR-positive area. **b** The ratio of fluorescence to EGFR over the entire tumor area was not significantly different between the LD and non-LD group (Scale bar: 5 mm). **c**–**e** Comparison of three metrics, including interquartile range (IQR), Pearson’s correlation coefficient between fluorescence and EGFR expression and CLCM correlation, all reflected less heterogeneity in antibody penetration across tumors in patients with loading dose (*p < 0.05; ns: *p* > 0.05). **f**, **g** Co-administration of parent antibody could overcome binding site barrier and improve antibody–dye distribution within the tumors. **f** As fluorescent antibody extravasates from blood vessels (red tube), it immediately binds EGFR in the tissue (represented by cells in the cube). **g** By co-administering non-fluorescent antibody, the same amount of fluorescent antibody reaches the tissue, but these antibodies compete for binding sites locally in the tissue. This results in improved distribution of fluorescent antibody at the microscopic scale (Scale bar: 250 μm). Mann–Whitney *U* test (two-tailed) was used in **b**–**e**. *p* value = 0.025 **c**, 0.025 **d**, 0.036 **e**. The number of independent patient samples available for **b**–**e**: *n* = 12 patients in the LD group and *n* = 10 in the non-LD group. Graphs plotted mean with standard deviation.

To quantitatively compare the antibody penetration between the two dosing cohorts, we divided each tumor into small square regions and measured the antibody penetration in each square region as the ratio of NIR fluorescence-positive area to EGFR-positive area, and plotted a heatmap to visualize its distribution (Fig. 5a). When quantifying the overall antibody penetration, as measured by the ratio of NIR to EGFR over the entire tumor area, no difference was found between the LD and non-LD dosing groups (Fig. 5b). However, when taking into account the micro-regional variations using the square regions, the IQR of region-based antibody penetration ratio across the tumor section was significantly lower in patients receiving a loading dose compared with the patients in the non-LD group (Fig. 5c) and the Pearson’s correlation coefficient between the fluorescence and the EGFR expression of the square regions across each tumor was significantly higher in the LD than the non-LD dose group (Fig. 5d). Moreover, the antibody penetration ratio of neighboring regions were significantly more correlated in tumors receiving the LD dose compared to tumors in the non-LD dose group (Fig. 5e). All these metrics indicated reduced heterogeneity of antibody distribution in the LD group. Furthermore, if we decrease the current square size by twofold, the antibody distribution in the LD is still significantly lower than the non-LD group (Supplementary Fig. 1a). However, if we increase the current square size by twofold, there is no statistically significant differences in antibody distribution per square between the LD and non-LD group any more (Supplementary Fig. 1c), suggesting that the improved distribution is “averaged over” and the squares measure the macroscopic distribution, which is not expected to differ.

In Fig. 5f, when only the antibody–dye conjugate was administered systemically: the antibody–dye extravasated from the blood vessel and diffused across the extracellular space to bind to and saturate the outer cell layers of the tumor nest, thereby forming a stalled saturation front (i.e., “binding site barrier”). However, in Fig. 5g, when the parent antibody was co-administered with the antibody–dye conjugate, the parent antibody diluted the antibody–dye conjugate and competed for binding sites in the outer cell layers of the tumor nest. As a result, the antibody–dye conjugate was able to penetrate deeper into the interior of the tumor nests, overcoming the “binding site barrier” and leading to improved antibody distribution in the tumor at the microscopic level despite minimal change at the macroscopic (e.g., whole tumor) scale.

### Slight downward trend in tumor uptake with increasing doses

**Fig. 6:**
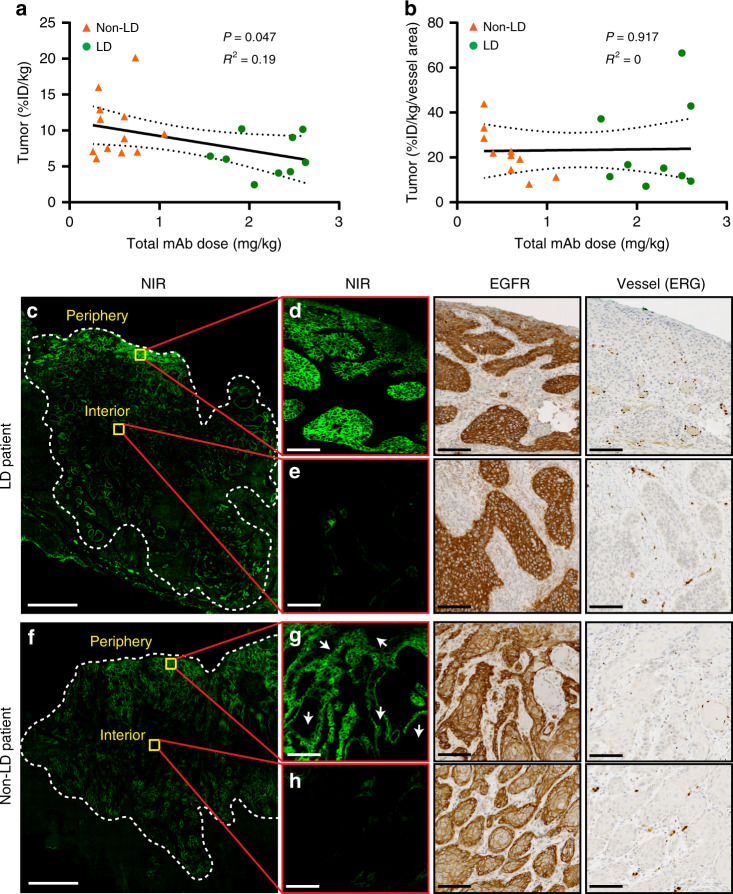
The impact of loading dose on total antibody uptake. **a** Tumor uptake (%ID kg^−^^1^) showed slightly decreasing trend with increasing total antibody dose. **b** Tumor uptake (%ID kg^−^^1^) normalized by vessel area fraction showed a constant trend with increasing total antibody dose, suggested the variations in vessel area between the two groups may have caused the downward trend in tumor uptake before normalization. **c–h** Examples from a LD patient and non-LD patient demonstrated partial saturation in the tumor periphery with loading dose. (Scale bar in **c** and **f**: 2 mm; scale bar in **d**, **e**, **g**, **h**: 100 μm). Linear regression was performed in **a**, **b** with the best-fit line (solid line), the 95% confidence bands (dotted line), goodness of fit (*R*^2^), and *p* value.

In preclinical models, tumor uptake measured by percent injected dose per gram (%ID g^−1^) was predicted to remain constant prior to saturation^13^. Consistent with this prediction, when sub-saturating doses of antibody–dye were delivered, the loading dose did not affect the MFI of both macroscopic and microscopic imaging. Although tumor uptake in %ID kg^−1^ showed a slight downward trend with higher doses of total antibody (Fig. 6a), this trend disappeared when normalizing by vessel area fraction (Fig. 6b), which suggests that this was caused by the variations in vessel density between the two groups. However, we have observed more frequent saturation of the tumor nests located at the tumor periphery in the LD group (Fig. 6c–e) compared with the non-LD group (Fig. 6f–h), suggesting an alternative possibility that regional saturation of tumor cell nests in the periphery allowed washout in this area while cells in the tumor core remained untargeted. Antibodies can theoretically diffuse ~1 mm before washing out of the tumor if they are in a saturated region with no binding sites^20^. Regional saturation in the tumor periphery of the LD group could cause a local decrease in %ID kg^−1^ as extravasated antibody encounters no free binding sites and washes out (Fig. 6c–e). In this case, the poorly vascularized regions in the center would remain unsaturated, continuing to take up more antibody with increasing total antibody doses (for a constant local %ID kg^−1^). (Note that in Fig. 6c–h, the low fluorescence “core” in the two tumors are macroscopic heterogeneities caused by vascularity differences^20,21^ rather than microscopic heterogeneities caused by binding site barrier.)

## Discussion

Poor tissue penetration has long been considered as a major hurdle for antibody-based cancer therapeutics^20^. The recent FDA approval of five ADCs in nine months provides new hope for realizing Paul Ehrlich’s magic bullet^22^. In particular, the multiple mechanisms of action of ADCs, from both the antibody and payload^23–25^, have the potential to drive strong immune responses and pair with immune checkpoint inhibitors (as seen with the promising 73.3% overall response rate (ORR) in urothelial carcinoma^26^). However, owing to dose-limiting toxicity of the small molecular payload, ADCs are usually administered at a much lower dose than their parent antibody and their efficacy is significantly limited by the binding site barrier that leads to inadequate tissue penetration. Several notable failures of ADCs have occurred over the past few years, but the recent success of four different ADCs for solid tumor indications is notable owing to the higher antibody doses in these regimens^26–29^. Presumably, higher antibody doses could increase tissue penetration, but current methods for imaging, such as positron emission tomography (PET) and single photon emission computed tomography (SPECT), do not have sufficient resolution to measure intratumoral antibody distribution in the clinic. Here, we use a NIR fluorescently labeled antibody, originally developed for intraoperative imaging^30^, to track the intratumoral distribution of antibody at single-cell resolution. We demonstrated that co-administration of the parent antibody with the antibody–dye conjugate significantly improved the distribution of the antibody–dye conjugate in human patients while lowering uptake in healthy tissue. Our previous data suggested that injecting unconjugated antibody can saturate the antibody sink, and prolong imaging time window^31^. However, there was no measurable benefit of using a loading dose in terms of increasing tumor-to-background ratio for surgical imaging. The current study shows that a loading dose can be beneficial in reducing the binding site barrier and increasing tissue penetration of antibody–dye conjugates, which supports the possibility for applying the same dosing strategy for ADCs that warrant further clinical investigation.

The macroscopic uptake of an antibody therapeutic at a given tumor region (e.g., tumor periphery vs interior) is driven by its vascularity or the permeability surface area product (independent of the loading dose)^2,32^, whereas the microscopic distribution is dependent on the amount of binding sites available for a given antibody^9^. Because antibodies bind to their targets much faster than they diffuse, their penetration in tumor tissue is severely limited (e.g., the “binding site barrier”^19^). Prior to a saturating dose of antibody (which was not achieved here), this limits the impact of a loading dose to improving penetration on the microscopic scale. By adding the loading dose, we are not changing the fluorescent antibody uptake in a given region, but we are blocking some binding sites so that fluorescent antibody can penetrate deeper into the tumor cell nest, overcoming the “binding site barrier” and leading to improved antibody distribution in the tumor at the microscopic level despite minimal change at the macroscopic (e.g., whole tumor) scale^13^. We also noted that given the large intratumoral heterogeneity of clinical tumors (e.g., the tumor morphology and vessel density), the improvements in distribution are more difficult to detect by eye than in more uniform preclinical animal models. Therefore, we developed image analysis techniques to quantify the relative changes of the intratumoral antibody distribution and penetration. Importantly, both preclinical^7^ and clinical studies^33,34^ showed that even when the improvement in microscopic distribution is hard to detect by eye, the improved antibody penetration still has significant impact in therapeutic efficacy. In theory, if the antibody dose is high enough, the antibody will reach all (accessible) binding sites within the tumor and saturate both the interior and periphery of the tumor, which has been shown in preclinical studies^6,13,35,36^. However, the limitation of doing a clinical study is that we cannot inject any dose of antibody–dye into the patients. With the clinically acceptable low doses of antibody–dye (consistent with ADC clinical dosing), we successfully demonstrated that statistically significant improvement of distribution and penetration can be detected at the microscopic scale, but not at the macroscopic scale. This proof-of-principle study is an important first step towards translating the dosing strategy into the clinic.

To quantify antibody distribution, we chose a more empirical approach rather than mechanistic analysis. Because of the limitations, such as fixed tissue requiring alignment of adjacent slices for EGFR, vessels and fluorescent antibody, and the heterogeneous morphology of the tissue, etc., a mechanistic modeling approach is challenging. Therefore, we opted for a more empirical approach (which uses more image processing and fewer model assumptions) to validate the improved microscopic distribution. Although a variety of quantitative metrics have been reported in the literature to measure tumor heterogeneity, there are no standardized metrics and the selection of heterogeneity measurements depends on the specific problem at hand^37,38^. For example, in this study, we chose IQR, which is defined as the 1st quartile subtracted from the 3rd quartile^39^, because it is a measure of statistical dispersion and can be used to describe the overall extent of the fluorescence intensity distribution (i.e., antibody–dye distribution); we chose entropy^40^ because it is a statistical measure of randomness/uncertainty in the image values and reflect the diversity of the fluorescence intensity distribution (i.e., antibody–dye distribution). We also used the uniformity because it measures the degree of uniformity or homogeneity in fluorescence intensity distribution^41^.

Although this study showed similarities with preclinical studies in terms of healthy tissue uptake, it showed a slightly decreasing trend in tumor uptake (%ID kg^−1^), which was different from preclinical prediction and suggested the inherent complexity in human tumors. One possibility is owing to variations in vessel density since the decreasing trend in tumor uptake disappeared after normalizing by vessel area fraction. An alternative hypothesis that could not be completely ruled out by the data is regional saturation of tumor cell nests in the periphery allowed washout in this area while cells in the tumor core remained untargeted, suggesting that both the microscopic distribution (binding site barrier) and macroscopic distribution (periphery vs interior) interact to determine the overall tumor uptake (%ID kg^−1^). Additional data, collected with different antigen targets in diverse tumor types, can shed light on this issue. Taken together, our study suggests that co-administration strategy would decrease off-target uptake and toxicity to healthy tissue^42,43^ while maintaining tumor uptake and improving distribution.

This study highlights the tumor distribution of therapeutic antibodies at clinically relevant doses (consistent with ADC dose) at subcellular resolution. Molecular imaging of radiolabeled antibodies has provided insight into the dosing and distribution on a macroscopic scale, but the inherent spatial limitations of PET and SPECT do not yield cellular resolution^44–46^. Autoradiography is another way to study the biodistribution of radiolabeled antibodies in biological samples. However, it has several limitations for measuring antibody microdistribution compared with fluorescently labeled antibodies^47–52^. These include the lack of cellular resolution (at typical specific activities and relevant antibody doses), the risk of exposing patients to radioactivity, challenges in quantification due to artifacts, and extremely long exposure times (e.g., for tritiated samples) required to accumulate enough signal (on the orders of days to months), etc. However, it is at the cellular level that antibodies engage their target, traffic through the endocytotic machinery, interact with immune cells, and generally perform their pharmacodynamic function. The cellular distribution is critical to this role as demonstrated by preclinical models. It is also consistent with clinical data, such as the increase in response rate of an anti-MUC16 ADC from 17% ORR to 45% ORR^33,34^ when the antibody dose was increased from 2.4 mg kg^−1^ to 5.2 mg kg^−1^ (although the drug to antibody ratio was decreased from 3.5 to 2, which maintained a similar cytotoxic payload dose). However, cellular distribution is typically not available during these trials. Therefore, the ability to measure this distribution in human tumors in early-phase clinical development, while technically challenging, could help advance the clinical translation of antibody-based therapeutics.

NIR fluorescence imaging is advancing as an intraoperative procedure to guide precision tumor resection^30,53–56^. Many of the same antibodies are used for imaging and therapy, providing a unique opportunity to track their distribution at the single-cell resolution. Although the antibody–dye conjugate (panitumumab-IRDye800CW) was used as a surrogate for ADC delivery, there are several similarities between the two: both panitumumab and trastuzumab (the parent antibody of trastuzumab emtansine and trastuzumab deruxtecan) bind to targets that belong to the ErbB family (EGFR/HER1 and HER2, respectively), and both receptor expression levels are high. Furthermore, the large size of the antibody generally controls the pharmacokinetics of antibody–dye and ADCs. Both drug conjugation and fluorescent labeling are often conducted with the goal of minimizing any impact on plasma clearance and distribution^17,57–59^.

This work shows that antibody dose can have a major impact on microscopic distribution. These differences would go undetected at the macroscopic level, such as via PET imaging. PET and SPECT provide non-invasive dynamic imaging and whole-body antibody distribution, but they cannot capture cellular detail. The macroscopic NIR imaging showed the same total tumor uptake of antibody, the metric captured by PET and SPECT given the several millimeter spatial resolution. In contrast, microscopic analysis indicated significant differences in the tissue and cellular distribution, below the resolution of clinical scanners but important for efficacy, suggesting the complementary nature of optical molecular imaging and nuclear imaging to measure antibody distribution.

In summary, given the safety of NIR imaging (no ionizing radiation), high sensitivity (detectable at therapeutic to sub-therapeutic doses), and ability to capture multiscale distribution within the tumor, NIR imaging could be a valuable tool in the development of antibody-based therapeutics. With the antibody–dye conjugates serving as a surrogate for ADCs, these results support the pursuit of higher antibody doses with ADCs to improve tissue penetration and expand the therapeutic window of ADCs.

## Methods

### Study design

A non-randomized prospective dose-ranging study was performed in patients with biopsy-proven HNSCC that were scheduled for surgical resection of the primary tumor for curative intent. The study protocol was approved by the Stanford University Institutional Review Board and the FDA (NCT 02415881), and written informed consent was obtained from all patients. The study was performed in accordance with the Helsinki Declaration of 1975 and its amendments, FDA’s ICH-GCP guidelines, and the laws and regulations of the United States. A total of 24 patients met eligibility criteria and were enrolled in this study between 09/2016-03/2018. Patients in this study received intravenous infusion of a total antibody dose of 0.3 mg kg^−1^ to 2.6 mg kg^−1^ (including both panitumumab and panitumumab-IRDye800CW) 1–5 days prior to surgery. More specifically, the loading dose cohort received an intravenous infusion of 100 mg of panitumumab followed by 0.5 mg kg^−1^ or 1 mg kg^−1^ of panitumumab-IRDye800CW, whereas patients in the non-loading dose cohort received an intravenous infusion of 25 mg or 50 mg of panitumumab-IRDye800CW. Specifically, panitumumab was intravenously administrated over 1 hour followed by a 15 min observation period. Thereafter panitumumab-IRDye800CW was intravenously administered over 1 hour also followed by 1 hour of monitoring. Because of the slow uptake of antibodies in tumor tissue and continual internalization by cells, very little antibody reaches the tumor prior to administration of the antibody–dye conjugate 15 minutes later. Therefore, the sequential administration of panitumumab and panitumumab-800CW is effectively co-administration.

The primary outcome of the clinical trial (NCT 02415881) was pre-defined as the safety profile of panitumumab-IRDye800CW. The secondary outcomes were pre-defined as (1) the efficacy of panitumumab-IRdye800CW to identify cancer compared with surrounding normal tissue and (2) the optimal timing of the surgical procedure to maximize the tumor to background ratio. These outcomes have been previously reported^16,30,31^. The current study is a retrospective ad hoc analysis using the clinical data set from the same clinical trial. The objective of the current study is to evaluate whether a loading dose of an unconjugated antibody could improve the microscopic distribution of an antibody–dye conjugate in a clinical setting.

### Antibody concentration quantification

The antibody concentration was measured from tumor and skin tissue samples obtained from study patients following a previously developed protocol^2^. In brief, tissue samples were homogenized in chilled lysis buffer containing a protease inhibitor (Roche, Indianapolis, IN), and then added into a 96-well plate for measurement by a NIR plate reader (Tecan, Zürich, Switzerland). The antibody concentration was extrapolated from a standard curve generated with control tissue homogenates and normalized by tissue weighted as ng mg^−1^ of tissue.

### Macroscopic fluorescence imaging analysis

The 5-mm thick tissue sections were imaged and quantified following a previously developed method^31^. In brief, after colocalizing the closed-field images of the 5-mm tissue sections with the H&E slides, region of Interest (ROIs) were drawn within the bulk tumor region in the closed-field images using imageJ and the integrated instrument software (Image Studio; LI-COR Biosciences, Lincoln, Nebraska, USA). All macro-sections of the primary tumors of each patient were analyzed. After the primary tumor was outlined, fluorescence intensity for all ROIs of the primary tumor were measured, and the tissue MFIs were defined by the following equation:

1 $${\mathrm{Tissue}}\,{\mathrm{MFI}} = 	\, (MFI1 \ast Area1 + MFI2 \ast Area2 + \cdots \\ 	+ MFIn \ast Arean)/(Area1 + Area2 + \cdots + Arean)$$

The fluorescence intensity distribution over the entire tumor region was also quantified with statistical metrics, including IQR^37^, entropy^2,38^, and uniformity^38,41^, which reflected the heterogeneity or homogeneity of antibody distribution at the macroscopic level.

### Microscopic fluorescence imaging analysis

The histology tissue section was processed and imaged following our previous protocol^2^. In brief, serial histology tissue sections were cut from a representative tissue block of each patient, with one slide stained for H&E, two slides immunohistologically stained with EGFR (pre-diluted), αSMA (dilution 1:1000), and ERG (ETS-related gene, a nuclear transcription factor stain for endothelial cells^60^; dilution 1:1000), and one slide for fluorescence microscopic imaging to characterize the microscopic antibody distribution. In brief, the micro-sections were dewaxed with xylene and rehydrated through gradient ethanol into water, followed by 4′, 6-diamidino-2-phenylindole nuclei staining. The whole-tumor region within each micro section was then scanned by a customized NIR fluorescence microscope (Leica Microsystems Inc. Chicago, IL) using the tile scan function with a NIR filter (excitation/emission: 774 nm/789 nm) at a resolution of 0.65 µm/pixel. Individual tiles were stitched into a single large mosaic image for subsequent analysis. The positive EGFR and αSMA staining area within the tumor region was identified through multilevel Otsu’s thresholding method^61^. The vessel area was represented by the area of the positive ERG staining, which was defined as above an intensity threshold of 50 as previously reported^2^.

Fluorescent antibody distribution area was segmented using a customized thresholding algorithms^2^. In brief, the intensity histogram of the tumor pixels were calculated and fitted into a “loglogistic” distribution (MATLAB function). Then a threshold was then experimentally identified based on the histogram distribution and a binary mask was generated with the threshold. Next, first-order statistical metrics, including mean, entropy, and IQR, were calculated from the segmented positive fluorescent tumor areas.

To quantify the antibody penetration into the tumor, the tumor region in a representative histology section of each patient was gridded into individual squares of *M* × *M* μm^2^ (*M* = 166, 333, 666). The antibody penetration degree within each tumor square was quantified by dividing the number of positive NIR fluorescent pixels by the number of EGFR-positive pixels. The overall distribution heterogeneity of each tumor was then measured by three ways: (1) IQR of the antibody penetration within all the squares, which has been previously proposed as a biologically relevant heterogeneity metric^37^; (2) Pearson’s correlation coefficient between the NIR fluorescence and EGFR expression per square across each tumor were computed for all the patients, which has been previously reported to be a suitable metric to show how much the antigen (EGFR) is targeted^62^ (insensitive to intensity differences); (3) a spatial metric^63^ was calculated by first computing the gray level correlation matrix of the NIR/EGFR per squares across each tumor, and then calculating the joint probability occurrence of the antibody penetration within each square to that of its neighboring squares, which can capture the microscopic variations of drug penetration.

### Statistical analysis and reproducibility

Patient clinical factors, including age (≤60, >60), gender, tumor site (tongue, other), tumor size (≤40 mm, >40 mm), and time to surgery (1–2 days, >3 days), were assessed between LD and non-LD groups with for the Mann–Whitney *U* test (continuous variables) and Fisher’s exact test (categorical variables). The intratumoral distribution pattern of antibody–dye were qualitatively assessed and compared between the LD and non-LD group. Quantitative metrics describing the uptake and distribution of the antibody–dye bioconjugate were derived from all the patients and compared between LD and non-LD groups with a Mann–Whitney *U* test. Descriptive statistics and figures were generated using GraphPad Prism (Version 8.4.1, GraphPad Software, La Jolla, CA, USA). All data are presented as mean or mean ± standard deviation. All statistical tests are two-sides with a *p* value of 0.05 or less considered as statistically significant. (*p < 0.05; **p < 0.01; ns not significant).

### Reporting summary

Further information on research design is available in the Nature Research Reporting Summary linked to this article.

## Supplementary information

Supplementary Information

Reporting Summary

## Data Availability

The source data underlying all main figures (Figs. 2, 3e, f, 4b–e, g–n, 5b–e, 6a, b) are provided as a Source Data file. The remaining data are available in the article, Supplementary Information, or available from the authors upon reasonable request. Source data are provided with this paper.

## Source data

Source Data
