# Supplementary material for: Co-administered antibody improves penetration of antibody–dye conjugate into human cancers with implications for antibody–drug conjugates
Source: Nat Commun. 2020 Nov 9;11:5667. doi: 10.1038/s41467-020-19498-y (PMC7652891; doi:10.1038/s41467-020-19498-y)

Supplementary Information

Co-administered Antibody Improves Penetration of Antibody-Dye Conjugate into Human Cancers with Implications for Antibody-Drug Conjugates

Lu et al.

Supplementary Fig. 1. The improvement in antibody distribution is only measurable at the microscopic level but not at the macroscopic level. If the grid size is decreased by twofold (**a**) compared to (**b**), the antibody distribution is still significantly better in the LD group than the non-LD group. However, if the square size is increased by twofold (**c**) compared to (**b**), the microscopic heterogeneity was averaged over and the difference is not measurable anymore. Tissue samples available for analysis in these patients are: n = 12 patients in the LD group; n = 10 patients in the non-LD group. Error bar means standard deviation. Mann-Whitney U test (two-sided) was used for statistical test. *$p < 0.05$; ns: $p > 0.05$. p value = 0.025 (**a**), 0.025 (**b**). Scale bar = 166µm in (**a**), 333µm in (**b**), 666 µm in (**c**).

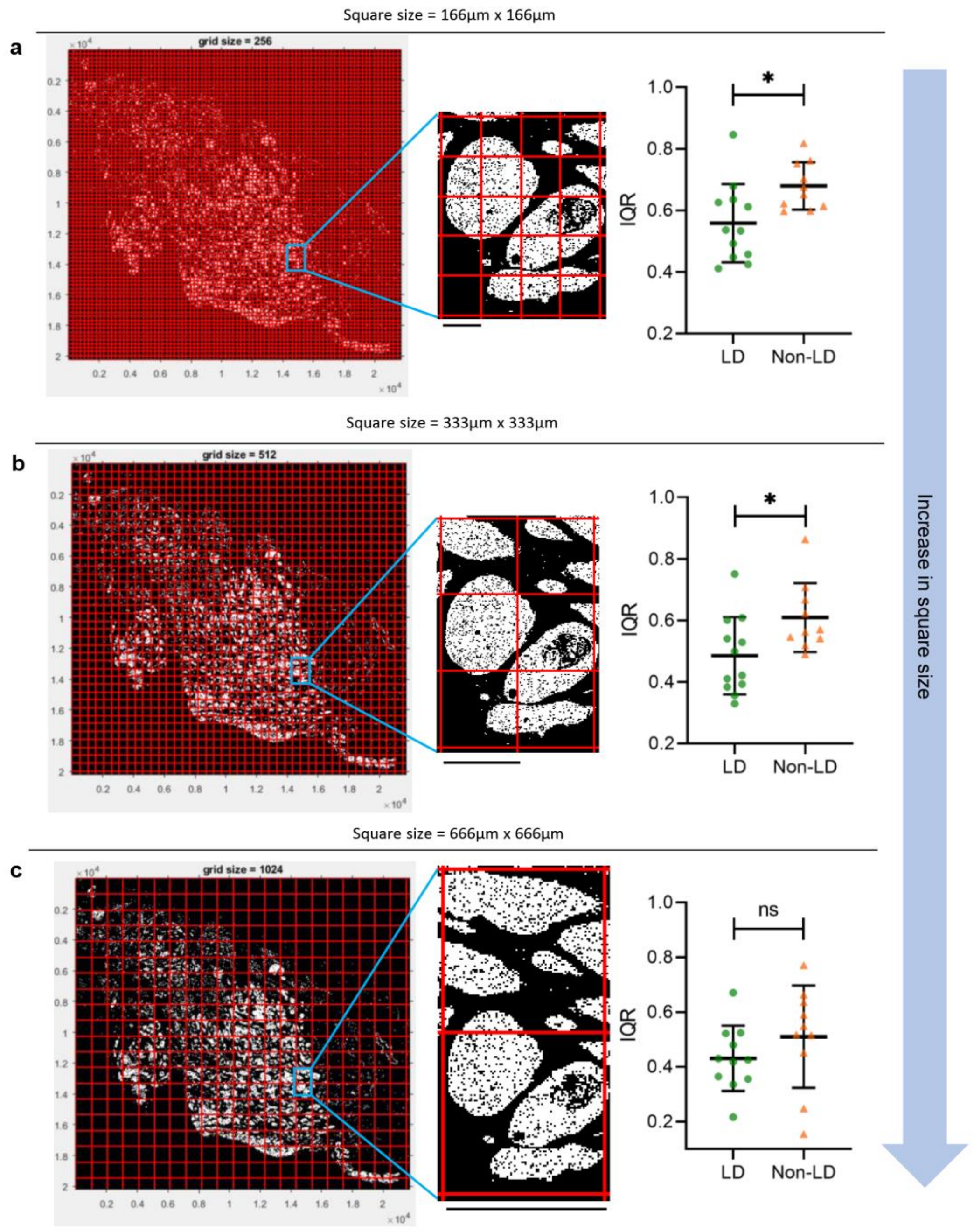

Supplement: Supplementary file 1 — Supplementary Information [file 41467_2020_19498_MOESM1_ESM.pdf]
