# Supplementary material for: Co-administered antibody improves penetration of antibody–dye conjugate into human cancers with implications for antibody–drug conjugates
Source: Nat Commun. 2020 Nov 9;11:5667. doi: 10.1038/s41467-020-19498-y (PMC7652891; doi:10.1038/s41467-020-19498-y)
Supplement: Supplementary file 2 — Reporting Summary [file 41467_2020_19498_MOESM2_ESM.pdf]

nature research

Corresponding author(s): Eben L. Rosenthal

Last updated by author(s): Sep 14, 2020

Reporting Summary

Nature Research wishes to improve the reproducibility of the work that we publish. This form provides structure for consistency and transparency in reporting. For further information on Nature Research policies, see our Editorial Policies and the Editorial Policy Checklist.

Statistics

For all statistical analyses, confirm that the following items are present in the figure legend, table legend, main text, or Methods section.

n/a	Confirmed	
☐	☒	The exact sample size (n) for each experimental group/condition, given as a discrete number and unit of measurement
☐	☒	A statement on whether measurements were taken from distinct samples or whether the same sample was measured repeatedly
☐	☒	The statistical test(s) used AND whether they are one- or two-sided<br>*Only common tests should be described solely by name; describe more complex techniques in the Methods section.*
☐	☒	A description of all covariates tested
☒	☐	A description of any assumptions or corrections, such as tests of normality and adjustment for multiple comparisons
☐	☒	A full description of the statistical parameters including central tendency (e.g. means) or other basic estimates (e.g. regression coefficient) AND variation (e.g. standard deviation) or associated estimates of uncertainty (e.g. confidence intervals)
☒	☐	For null hypothesis testing, the test statistic (e.g. F, t, r) with confidence intervals, effect sizes, degrees of freedom and P value noted<br>*Give P values as exact values whenever suitable.*
☒	☐	For Bayesian analysis, information on the choice of priors and Markov chain Monte Carlo settings
☒	☐	For hierarchical and complex designs, identification of the appropriate level for tests and full reporting of outcomes
☐	☒	Estimates of effect sizes (e.g. Cohen's d, Pearson's r), indicating how they were calculated

Our web collection on statistics for biologists contains articles on many of the points above.

Software and code

Policy information about availability of computer code

Data collection	The macroscopic fluorescence images of the primary tumor specimens were collected by using Image Studio version 5.2; The microscopic fluorescence images were acquired by using Leica Application Suite X 3.0.2.16120; The plate reader data were measured by using the Tecan SPARKCONTROL v2.1.
Data analysis	Statistical analysis: Graphpad Prism (Version 8.4.1, GraphPad Software, La Jolla, CA, US); Image analysis: Matlab (version 2020a, MathWorks, Natick, MA); imageJ version 1.52k; Image Studio version 5.2

For manuscripts utilizing custom algorithms or software that are central to the research but not yet described in published literature, software must be made available to editors and reviewers. We strongly encourage code deposition in a community repository (e.g. GitHub). See the Nature Research guidelines for submitting code & software for further information.

Data

Policy information about availability of data

All manuscripts must include a data availability statement. This statement should provide the following information, where applicable:

- Accession codes, unique identifiers, or web links for publicly available datasets
- A list of figures that have associated raw data
- A description of any restrictions on data availability

The source data underlying all main figures (Fig.2, Fig.3e-f, Fig.4b-e,g-n, Fig.5b-e, Fig.6a-b) are provided as a Source Data file. Additional data supporting the findings in this study are available from the corresponding author upon reasonable request.

Field-specific reporting

Please select the one below that is the best fit for your research. If you are not sure, read the appropriate sections before making your selection.

☒ Life sciences ☐ Behavioural & social sciences ☐ Ecological, evolutionary & environmental sciences

For a reference copy of the document with all sections, see nature.com/documents/nr-reporting-summary-flat.pdf

Life sciences study design

All studies must disclose on these points even when the disclosure is negative.

Sample size	No sample-size calculation was performed. Sample size was chosen based on the number of patients included in our phase I study, at the time 24 patients. The sample size was considered sufficient for data evaluation.
Data exclusions	None.
Replication	The fluorescence uptake and distribution in tumors were measured and quantified in multiple different ways. The comparison between the loading and non-loading groups were also conducted at both macroscopic and microscopic level. These analysis showed consistent results and indicated our analysis is reproducible.
Randomization	This is a single-armed first-in-human clinical study.
Blinding	This is an open-label first-in-human clinical study.

Reporting for specific materials, systems and methods

We require information from authors about some types of materials, experimental systems and methods used in many studies. Here, indicate whether each material, system or method listed is relevant to your study. If you are not sure if a list item applies to your research, read the appropriate section before selecting a response.

Materials & experimental systems

n/a	Involved in the study	
☐	☒	Antibodies
☒	☐	Eukaryotic cell lines
☒	☐	Palaeontology and archaeology
☒	☐	Animals and other organisms
☐	☒	Human research participants
☐	☒	Clinical data
☒	☐	Dual use research of concern

Methods

n/a	Involved in the study	
☒	☐	ChIP-seq
☒	☐	Flow cytometry
☒	☐	MRI-based neuroimaging

Antibodies

Antibodies used	panitumumab-IRDye800CW (produced through NCI's NEXT program);<br>anti-EGFR antibody (clone EP38Y; lot no. 2111RQ1802C; ThermoFisher Scientific, Waltham, MA);<br>anti-α-SMA mouse monoclonal antibody (clone: 1A4, code: M0851, DAKO);<br>anti-ERG rabbit monoclonal antibody (clone: EPR3864, cat #: ab92513, Abcam);
Validation	Panitumumab-IRDye800CW was produced through NCI's NEXT program. Quality control included analysis of drug product in sterile vials for particulates and integrity of the sterilizing filter. Sterile via were transported to Stanford University under temperature controlled conditions. Vials were stored at Stanford, at the Stanford University Medical Center Investigational Pharmacy. A certificate of analysis was provided to us by NCI and stability tests are/were regularly performed to confirm product stability.<br><br>anti-EGFR antibody: https://assets.thermofisher.com/TFS-Assets/APD/Specification-Sheets/D12446~.pdf<br>anti-α-SMA antibody: https://www.agilent.com/en/product/immunohistochemistry/antibodies-controls/primary-antibodies/actin-(smooth-muscle)-(concentrate)-76542<br>anti-ERG antibody:https://www.abcam.com/erg-antibody-epr3864-ab92513.html

Human research participants

Policy information about studies involving human research participants

Population characteristics	Patients, M/F, >19 years of age, with biopsy-proven head and neck squamous cell carcinoma scheduled to undergo surgical resection of curative intent were eligible to participate in the study. A total of 24 patients were enrolled in the study. The

	characteristics of patients are: Mean age 62 years (range 32-85 years(, and the majority presented with oral squamous cell carcinoma (88%). The average time of infusion to the start of surgery was 2.5 days (range 1-5).
Recruitment	Patients were recruited from the head and neck oncology practice at Stanford. All patients were evaluated by Dr. Rosenthal and a medical-oncologist (Dr. Colevas) to ensure that the patient met all eligibility criteria. Patients were initially approached by the protocol director, Dr. Rosenthal.<br><br>Written Informed Consent and HIPAA Authorization were obtained after the Informed Consent was reviewed and the study was fully explained to the patient including potential risks and discomforts – this was done by the clinical trial coordinators (Mr. Oberhelman and/or Mrs. Chirita). Full disclosure of the details and the investigational nature of the proposed protocols was provided by both the protocol director, Dr. Rosenthal, and by the clinical trial coordinators.<br><br>The protocol adhered to regulations to provide protection for human subjects in clinical investigations described by the general requirements for informed consent. The DSMB of Stanford University provided oversight for the trial and trial safety.<br><br>Bias: To the best of our knowledge, self-selection bias did not occur.
Ethics oversight	Stanford University's IRB, the Administrative Panel on Human Subjects in Medical Research

Note that full information on the approval of the study protocol must also be provided in the manuscript.

Clinical data

Policy information about clinical studies

All manuscripts should comply with the ICMJE guidelines for publication of clinical research and a completed CONSORT checklist must be included with all submissions.

Clinical trial registration	NCT02415881
Study protocol	The study protocol (Stanford IRB-35064) is available from the corresponding author upon reasonable request.
Data collection	Enrolled patients underwent surgery between 9/2016-3/2018. Immediately after surgical resection, fresh tissue samples were obtained from each patient when available and antibody concentration was quantified using these tissue samples. After formalin-fixation, the primary tumor specimen was breadloafed into about 5 mm thick tissue sections and imaged with a closed-field fluorescence imaging system. Subsequently, the thick tissue sections were paraffin embedded into tissue blocks and then serially sectioned at 5 µm thickness (histology sections) for near-infrared fluorescence microscopic imaging and histopathology assessment (hematoxylin and eosin (H&E) slides). All these data were collected in the Rosenthal Laboratory at Stanford University. Data analysis, including fluorescence imaging data analysis, immunohistochemistry and statistical analysis was performed between 1/2020 and 8/2020 in the Rosenthal Laboratory at Stanford University.
Outcomes	The primary outcome of the clinical trial (NCT 02415881) was pre-defined as the safety profile of panitumumab-IRDye800CW. The secondary outcomes were predefined as (1) the efficacy of panitumumab-IRdye800CW to identify cancer compared to surrounding normal tissue and (2) the optimal timing of the surgical procedure to maximize the tumor to background ratio. These outcomes were previously reported: [1]Gao RW, et al. Theranostics. 2018;8(9):2488-2495. [2] Gao RW, et al. Cancer Res. 2018;78(17):5144-5154. [3]Nishio N, et al. Mol Imaging Biol. 2020;22(1):156-164.)<br><br>The current study is a retrospective ad-hoc analysis using the clinical dataset from the same clinical trial (NCT 02415881). The objective of the current study is to evaluate whether a loading dose of an unconjugated antibody could improve the intratumoral distribution of an antibody-dye conjugate in a clinical setting.
